# Postoperative Pain and Opiate Requirement is Increased Following Second-Side Surgery Among Patients Undergoing Staged Total Knee Arthroplasty

**DOI:** 10.1016/j.artd.2024.101591

**Published:** 2024-12-14

**Authors:** Vivek P. Chadayammuri, Shuvalaxmi D. Haselton, Elizabeth Diaz, Roger H. Emerson

**Affiliations:** Texas Center for Joint Replacement, Texas Health Physicians Group, Plano, TX, USA

**Keywords:** Analgesia, Postoperative pain, Bilateral, Staged total knee arthroplasty

## Abstract

**Background:**

Primary total knee arthroplasty (TKA) continues to grow exponentially, with a significant subset of patients requiring staged bilateral procedures. The optimal interval between staged procedures and effective strategies to minimize postoperative pain to enhance rehabilitation and mobility remain poorly understood.

**Methods:**

160 consecutive patients undergoing staged bilateral TKA between August 2017 and January-2021 was retrospectively reviewed. Patients with a history of chronic opioid dependency were excluded. Baseline demographics, primary outcome measures, including visual analog scale (VAS) pain scores, perioperative opioid utilization (MME/day), and surgical satisfaction were evaluated. All patients had a minimum follow-up of 1-year-postoperatively. All univariate and multivariate statistical analyses were performed with significance given by *P* < .05.

**Results:**

The mean interval between staged TKA was 8-weeks (standard deviation, 4.9-weeks). Preoperative VAS pain scores were significantly higher for the initial TKA, confirming that the more symptomatic knee was addressed first. Despite this, VAS pain scores were significantly increased following second-side TKA at 6-months postoperatively (*P* = .001). Multivariate analysis identified weekly interval duration between staged procedures as the single-most predictive factor of increased pain following second-side TKA (β = −0.106; *P* < .01). Female patients were increasingly susceptible to elevated pain levels following second-side TKA (β = 0.372; *P* = .057).

**Conclusions:**

Postoperative pain increases after second-side TKA in staged-bilateral procedures, despite the more symptomatic side being addressed first. Our study identified weekly interval between staged procedures as the single-most predictive factor of pain, and female patients being predisposed to heightened pain following second-side TKA; implicating nociceptive pathways require weeks to normalize, necessitating gender-specific pain management and extended intervals.

## Introduction

In the United States, approximately 55 million adults are affected by osteoarthritis (OA) of the hip or knee, a number projected to increase in line with rising average life expectancy and population size [1,2]. This demographic trend underscores the escalating demand for total knee arthroplasty (TKA), with epidemiological studies projecting an annual volume of 3 million TKAs by 2040, up from 700,000 in 2012 [1,2]. Notably, a significant proportion of these cases are bilateral and tend to be performed in a staged manner, beginning with the more symptomatic side.

TKA is consistently associated with higher levels of postoperative pain and a longer rehabilitation course compared to hip and shoulder arthroplasty [3,4]. Furthermore, in patients undergoing bilateral staged TKA, the second-side surgery tends to result in increased postoperative pain, regardless of the Kellgren-Lawrence OA grade [5]. Potential strategies to mitigate this phenomenon are crucial, as increased postoperative pain is widely recognized as one of the strongest prognostic indicators of mobility, ambulation, and overall patient satisfaction following TKA. Indeed, an international, multicenter study reported that 38% of patients undergoing primary TKA experience inadequate postoperative pain control, directly impacting their surgical function and satisfaction [6].

The purpose of this study was to characterize pain severity, postoperative opiate utilization, and potential prognostic factors in a consecutive cohort of patients undergoing primary, staged TKA at a single high-volume, total joint replacement center. The hypothesis was that a shorter interval duration between staged surgeries and a higher patient body mass index (BMI) would be the primary predictive variables associated with increased postoperative pain following second-side TKA.

## Material and methods

### Study design and sample population

Institutional review board approval was established to prospectively collect subjective and objective clinical outcome measures on patients undergoing primary total hip and TKA arthroplasty at a single high-volume dedicated total joint replacement center at a tertiary care hospital. A consecutive cohort of patients undergoing primary, elective, staged bilateral TKA between August 2017 and January 2021 with a minimum of 1 year postoperative follow-up was queried and considered eligible for inclusion in the present study. Patients were excluded given any of the following conditions: (1) nonelective procedure, (2) simultaneous bilateral TKA under a single anesthetic session, (3) revision TKA, or (4) tumor-related arthroplasty procedures. Additionally, patients who underwent arthroplasty procedures on a different, non-knee joint (eg, shoulder) within 12 months of their primary TKA were excluded to minimize potential confounding of reported pain scores.

The following demographic characteristics were abstracted for all patients: age, gender, BMI, American Society of Anesthesiologist physical classification score, and history of various comorbidities (diabetes, renal/hepatic impairment, cancer, rheumatoid arthritis, chronic back pain, and depression). BMI (kg/m^2^) was interpreted as underweight being less than 18.0; normal weight, 18.5-24.99; overweight, 25-29.99; or obese, 30 or greater. Type and amount of postoperative opiate utilized was recorded and converted to morphine milligram equivalents per day (MMEs/day). The total morphine milligram equivalent quantity was calculated for each patient using conversion factors of 0.1 for each mg tramadol and 1.5 for every mg of oxycodone, up to 3 months after surgery. In all cases, the amount of prescribed and filled scheduled controlled substance pain medications was confirmed using the Texas Prescription Monitoring Program.

### Surgical technique and analgesia

A standardized multimodal pain regimen was implemented at pre-, intra-, and postoperative phases of care for all patients undergoing primary elective TKA. Preoperatively, this involved administration of acetaminophen 975 mg, celecoxib 200 mg, and gabapentin 300 mg by mouth (PO). All surgeries are performed with a combination of general anesthesia in conjunction with ultrasound-guided saphenous nerve (adductor canal) block involving a 10-mL mixture of 0.5% ropivacaine. A periarticular local anesthetic injection was also administered, comprised of 30 mg/mL ketorolac (Toradol was contraindicated for patients with active peptic ulcer disease, a recent history of acute gastric bleeding, or chronic kidney disease classified as stage 3 or higher), 0.25 mL of 1-mg/mL epinephrine, 40-mL 0.9% normal saline, and 30-ml liposomal bupivacaine (Exparel; Pacira Pharmaceuticals, Inc., Parsipanny, NJ). Postoperatively, patients were treated with acetaminophen 975 mg PO every 8 hours (q8h), meloxicam 7.5 mg PO daily (QD) (Toradol was contraindicated for patients with active peptic ulcer disease, a recent history of acute gastric bleeding, or chronic kidney disease classified as stage 3 or higher), methocarbamol 500 mg PO three times daily, and tramadol 50 mg every 4 hours (q4h) PRN for severe pain. Oxycodone 5 mg PO every 6 hours (q6h) was also prescribed for breakthrough pain only.

Patients were admitted for overnight stay and initiated on a standard physical therapy program while in the hospital. Well-trained physical therapists associated with our total joint replacement program implemented a standard rehabilitation protocol, involving ambulation the day of the surgery and mobilization of halls and stairs the following day preceding discharge to home. All patients were instructed to maintain a protected gait using a walker for the first 2 weeks postoperatively and subsequently transitioned to ambulation with a cane for the following 2 weeks postoperatively. Progression to unassisted gait was initiated once complete resolution of pain in the operative extremity and satisfactory gait mechanics were observed, as assessed during an in-office evaluation. This typically occurred between 4 and 6 weeks postoperatively. Physical therapy was generally implemented for a total of 6 weeks postoperatively.

### Statistical analysis

A priori power analysis assuming a small effect size (0.25), alpha level of 0.05, power of 0.80, and inclusion of up to 5 explanatory variables determined that a minimum sample size of 128 patients would be necessary (G∗Power 3.1, Christian-Albrechts-Universität, Kiel, Germany). Accounting for a 20% attrition rate due to incompleteness of longitudinal retention data, the final required sample size was computed to be 160 patients.

Descriptive statistics were reported as means and standard deviations for quantitative variables and as counts and frequencies for categorical variables. All variables were evaluated for distribution of normality using a combination of histograms, Q-Q plots, and the Shapiro-Wilk tests (normality indicated by *P* > .05). Univariate comparisons of continuous variables (pain score, total MME/day, and Knee Society Score (KSS)) between staged procedures were evaluated using the paired-samples *t* test. Univariate comparisons of the proportion of patients requiring opiate medication at various time intervals postoperatively between staged procedures was evaluated using McNemar’s test. A stepwise multiple linear regression procedure was performed to evaluate whether any significant (*P* < .05) or near-significant (*P* < .10) factors from univariate analyses served as independent predictors of increased visual analog scale (VAS) pain scores following second-side surgery. Significance for all comparisons was set at *P* < .05 (2-tailed). All analyses were conducted using IBM SPSS Statistics Version 29.0 (Statistical Package for the Social Sciences, Chicago, IL) and SAS Statistical Software Version 9.4 (SAS Institute Inc., Cary, NC).

## Results

### Participants and descriptive data

The final study cohort was comprised of 160 patients (62 males, 78 females) with a mean age of 67.5 years (standard deviation (SD), 8.2 years) and mean BMI of 34.9 kg/m^2^ (SD, 8.8 kg/m^2^). Forty-six (26.7%) patients had type 2 diabetes. The mean interval between staged (second-side) TKA was 8.3 weeks (SD, 4.9 weeks). Additional baseline characteristics are summarized in Table 1.

**Table 1:** Baseline demographic profile of patients undergoing staged bilateral TKA (N = 160).

Age, mean (SD), y	67.5 (8.2)
BMI, mean (SD), kg/m^2^	34.9 (8.8)
Male, n (%)	62 (38.8)
Diabetic, n (%)	46 (26.7)
Mean duration of interval between staged TKA, mean (SD), wk	8.3 (4.9)

### Quantitative metrics of pain between staged bilateral TKA

**Figure 1:**
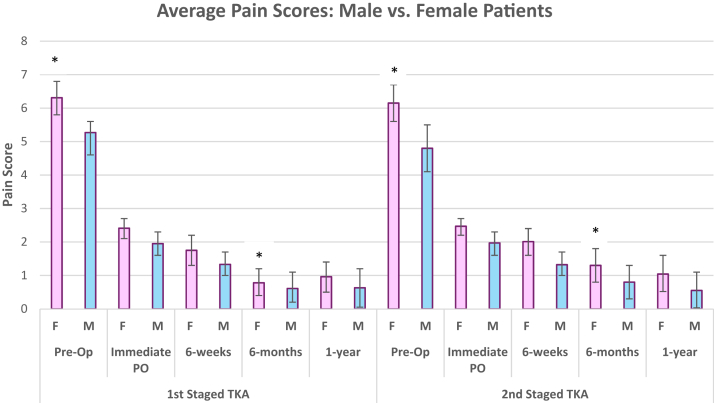
Mean pain scores in male and female patients for 1st and 2nd staged TKA. Error bars represent 95% CI (∗ = statistically significant). CI, confidence interval.

Preoperative (baseline) VAS pain scores were decreased approximately 1 point (*P* < .003) for second-side surgery, implying that the more symptomatic knee was appropriately identified and operated on first. A statistically significant increase in VAS pain scores was noted following second-side surgery at 6-months postoperatively (0.66 vs 1.08, *P* < .001), despite equivalent opiate utilization (48.5 vs 51.6 MME/day, *P* = .326; Table 2, Figure 1). The proportion of patients with requirement for prolonged opiate treatment of postoperative pain at 6 weeks postoperatively remained consistent between first- and second-side surgeries (5.6% vs 6.3%, *P* > .5). KSS scores surveyed 1 year postoperatively did not vary significantly between staged TKA (mean difference = 0.24; *P* = .207; Table 2).

**Table 2:** Pain scores and opiate utilization among patients undergoing staged bilateral TKA.

Metric	First TKA	Second (staged) TKA	*P* value
Pain score, mean (SD)			
Preop	6.13 (2.47)	5.82 (2.47)	**.003**
2 wk postop	2.18 (1.47)	2.22 (1.44)	.727
6 mo postop	0.66 (1.67)	1.08 (2.11)	**.001**
1 y postop	0.75 (1.77)	0.82 (1.93)	.666
Patients on opiate meds at 6 wk postop, n (%)	9 (5.6)	10 (6.3)	1.000
Total MME/day	48.5 (39.2)	51.6 (43.5)	.326
KSS score at 1 y postop	67.31 (18.07)	67.08 (17.99)	.207

Bold values indicate significant results.

### Independent factors predictive of increased pain at 6 weeks postoperatively followed second-side, staged TKA

Multivariate regression analysis demonstrated that the interval between staged TKA was the single-most important predictor of increased VAS pain score at 6 weeks postoperatively following second-side (staged) TKA, with each incremental weekly increase between staged procedures conferring improvement in pain tolerance (β coefficient −0.106; 95% confidence interval, −0.17 to −0.5; *P* < .01, Table 3). Female gender achieved near-statistical significance as a predictive factor of increased pain following second-side TKA also (β coefficient 0.372; 95% confidence interval, 0.02-0.76; *P* = .057, Table 3). These independent predictive variables outweighed the effect of all other demographic variables, including age and BMI.

**Table 3:** Multiple regression model of predictive variables for increased pain score at 6 mo after second (staged) TKA.

	Unstandardized b coefficient	95% CI	*P* value
Interval between staged TKA (wk)	0.106	−0.17 to −0.05	**<.01**
Gender (female)	0.372	−0.01 to 0.76	.057^a^

CI, confidence interval.

F (5,122) = 3.317, *P* = .008; adj. R^2^ = 0.084.

Bold values indicate significant results.

a Denotes near-significant trend (.05 < *P* < .10).

## Discussion

The rapidly rising prevalence of knee OA continues to drive an increasing demand for primary elective TKA, with recent epidemiological projections forecasting a 469% rise by 2060 within the United States alone [2]. A significant subset of these patients present with bilateral knee OA due to a combination of genetic predisposition, anatomic misalignment, and aberrant gait kinematics [[7], [8], [9], [10]]. Indeed, Metcalfe et al. observed that 26% of adults experiencing debilitating knee pain have bilateral OA and approximately 80% of those with unilateral OA progress to bilateral OA within a 12-year timeframe [8]. Hence, an understanding postoperative pain dynamics and potential prognostic factors in patients undergoing staged bilateral TKA is of critical interest.

The results of our study demonstrate that second-side surgery in patients undergoing bilateral, staged primary TKA confers a marked increase in postoperative VAS pain scores and MME/day utilization of controlled substance pain medications compared to initial-side surgery, with normalization (equivalence) of this trend only at 1 year postoperatively. Of note, preoperative (baseline) VAS pain scores were modestly decreased for second-side surgery, indicating that the more symptomatic side was routinely identified and preferentially operated upon first. Consistent with our findings, a prospective study involving 35 patients undergoing bilateral staged TKA by Sun et al. also identified significantly higher VAS pain scores following the second TKA, although their study implemented a 1-week interval between staged procedures compared to the average 8-week interval in our study [5]. Additionally, a retrospective review of 407 patients undergoing staged bilateral TKA by Memtsoudis et al. observed a 6% increase in opiate demand when the interval between staged TKAs was less than 3 months, compared to a 4% increase from first-side surgery when the interval ranged between 3 and 6 months [11].

Taken together, these findings underscore the need for thorough preoperative counseling to set realistic patient expectations among patients undergoing bilateral, staged TKA. Multivariate analysis identified that a longer interval duration between staged procedures was the single-most protective factor against exacerbated pain following second-side surgery. We propose that this finding is due to a "second-hit phenomenon," similar to that observed in patients with polytrauma underlying the emergence of 'damage control orthopaedics' principles. Specifically, it has been shown a systemic hyperinflammatory response is triggered by the initial injury and subsequently exacerbated by early surgical intervention, leading to potentially dire complications. [12]. By comparison, elevated levels of proinflammatory cytokines such as interleukin-1-beta, interleukin-6, and tumor necrosis factor alpha (TNF-α)—frequently elevated following primary TKA—have also been implicated in central sensitization of spinal cord dorsal horn neurons, contributing to the development of hyperalgesia and chronic pain [13,14]. An insufficient interval for the normalization of proinflammatory markers between staged bilateral procedures could lead to elevated levels due to the additional insult of second-side surgery, resulting in heightened postoperative pain. Of note, it is likely that peripheral nociceptive pathways are also implicated in the heightened postoperative pain following staged TKA, as prior studies have shown an incomplete resolution of postoperative pain with low-dose ketamine infusion—a known inhibitor of central sensitization nociceptive pathways—in staged TKA contexts [3]. Although our study was not designed to identify an exact interval threshold between staged TKA procedures for normalization of postoperative pain, multivariate analysis demonstrated that each additional week between staged procedures conferred a statistically significant reduction in VAS pain scores following second-stage TKA.

Another notable finding from our study was that female patients exhibited a near-significant increase in postoperative pain following second-side TKA compared to their male counterparts (*P* = .057). While literature specifically assessing gender-specific differences in pain perception following staged bilateral TKA is rather limited, previous studies have consistently demonstrated higher postoperative pain scores among female patients up to 6 weeks following unilateral TKA [15]. These gender differences are thought to derive from a combination of factors, including hormonal influences, pain reporting tendencies, and neuromuscular variances. Notably, female patients are known to harbor higher baseline levels of proinflammatory cytokines such as interleukin-6 and tumor necrosis factor alpha [16] and estrogen levels that are linked to hyperalgesia via N-methyl-D-aspartate and mitogen-activated protein kinase-mediated nociceptive pathways [17]. Additionally, a prospective study of 40 patients undergoing unilateral TKA by Sydney-Mackler et al. revealed significantly reduced quadriceps strength and poorer performance on timed up and go and stair-climb tests among female patients, a trend that normalized by 2 months postoperatively [18]. Taken together without our clinical findings, it appears that consideration of gender-specific pain management strategies and neuromuscular variances in the acute postoperative period, alongside extended intervals between staged bilateral TKA procedures, is optimal for mitigation of heightened postoperative pain states that may occur following second-stage TKA.

Strengths of our study include its relatively large sample size of patients undergoing staged bilateral TKA and rigorous monitoring of postoperative pain metrics. Indeed, the latter was facilitated by the development of a unique electronic medical record module in collaboration with our hospital information technology specialists for precise tracking. However, a few key limitations must be acknowledged. Firstly, the mean interval duration between staged TKA procedures was 8 weeks in our study and had marginal variability, thereby restricting the ability to determine an optimal interval threshold between staged TKA procedures to achieve normalization of postoperative pain. Additionally, the urban-based nature of the patient cohort may limit the generalizability of the findings to other demographic or geographical contexts. Indeed, prior studies have reported an increased incidence of acute postoperative complications such as deep vein thrombosis/pulmonary embolism and acute prosthetic joint infection in association with shorter intervals between staged bilateral TKA, a phenomenon that was not observed in our study. This is potentially due to differing baseline clinical and functional characteristics of the sampled patient cohorts [19]. Also, our study excluded patients with pre-existing chronic opiate dependence in order reduce risk of confounding. Finally, the observational (clinical) nature of our study precludes a mechanistic understanding of the heightened pain response following second-side surgery and among female patients; additional basic and translational studies are needed for this. Nonetheless, we believe that the presented study highlights important clinical considerations for patients undergoing staged bilateral TKA, including the potential benefits of extending the interval between staged procedures and adopting gender-specific pain management strategies to optimize patient outcomes.

## Conclusions

Increased post-operative pain after second-side TKA in staged bilateral procedures is observed despite the more symptomatic knee being addressed first. This study identified that the weekly interval between staged procedures is the single-most predictive factor of increased post-operative pain. Additionally, female patients are at a higher risk of being predisposed to heightened pain following second-side TKA, which implies nociceptive pathways require weeks to normalize. This necessitates extended intervals between staged procedures and gender-specific pain management methods.

## Conflicts of interest

The authors declare there are no conflicts of interest.

For full disclosure statements refer to https://doi.org/10.1016/j.artd.2024.101591.

## CRediT authorship contribution statement

**Vivek P. Chadayammuri:** Writing – review & editing, Writing – original draft, Formal analysis. **Shuvalaxmi D. Haselton:** Writing – original draft, Project administration, Formal analysis, Data curation. **Elizabeth Diaz:** Writing – review & editing, Writing – original draft, Conceptualization. **Roger H. Emerson:** Writing – review & editing, Supervision, Project administration, Conceptualization.

## Appendix A Supplementary data

Conflict of Interest Statement for Diaz

Conflict of Interest Statement for Emerson

Conflict of Interest Statement for Haselton

Conflict of Interest Statement for Chadayammuri

## References

[1] Inacio M.C.S., Paxton E.W., Graves S.E., Namba R.S., Nemes S. (2017). Projected increase in total knee arthroplasty in the United States – an alternative projection model. Osteoarthritis Cartilage.

[2] Shichman I., Roof M., Askew N., Nherera L., Rozell J.C., Seyler T.M. (2023). Projections and epidemiology of primary hip and knee arthroplasty in medicare patients to 2040-2060. JB JS open access.

[3] Jung Koh H., In Y., Kim E.S., Hwang J.W., Kim J.Y., Lim S.J. (2020). Does central sensitization affect hyperalgesia after staged bilateral total knee arthroplasty? A randomized controlled trial. J Int Med Res.

[4] Christensen T.H., Gemayel A.C., Bieganowski T., Lawrence K.W., Rozell J.C., Macaulay W. (2023). Opioid use during hospitalization following total knee arthroplasty: trends in consumption from 2016 to 2021. J Arthroplasty.

[5] Sun J., Li L., Yuan S., Zhou Y., Lai Y.H. (2015). Analysis of early postoperative pain in the first and second knee in staged bilateral total knee arthroplasty: a retrospective controlled study. PLoS One.

[6] Connelly J.W., Galea V.P., Rojanasopondist P., Nielsen C.S., Bragdon C.R., Kappel A. (2020). Which preoperative factors are associated with not attaining acceptable levels of pain and function after TKA? Findings from an international multicenter study. Clin Orthop Relat Res.

[7] Metcalfe A., Stewart C., Postans N., Dodds A., Smith H., Holt C.A. (2012). Biomechanics of the unaffected joints in patients with knee osteoarthritis. Orthopaedic Proceedings.

[8] Metcalfe A.J., Andersson M.L., Goodfellow R., Thorstensson C.A. (2012). Is knee osteoarthritis a symmetrical disease? Analysis of a 12 year prospective cohort study. BMC Muscoskel Disord.

[9] Sharma L., Lou C., Cahue S., Dunlop D. (2000). The mechanism of the effect of obesity in knee osteoarthritis: the mediating role of malalignment. Arthritis Rheum.

[10] Valdes A.M., McWilliams D., Arden N.K., Doherty S.A., Wheeler M., Muir K.R. (2010). Involvement of different risk factors in clinically severe large joint osteoarthritis according to the presence of hand interphalangeal nodes. Arthritis Rheum.

[11] Wilson L., Fiasconaro M., Liu J., Poeran J., Poultsides L., Memtsoudis S.G. (2020). Risk of chronic opioid use after simultaneous versus staged bilateral knee arthroplasty. Reg Anesth Pain Med.

[12] Robert C.S., Pape H.C., Jones A.L., Malkani A.L., Rodriguez J.L., Giannoudis P.V. (2005). Damage Control Orthopedics: evolving concepts in the treatment of patients who have sustained orthopedic trauma. Instr Course Lect.

[13] Kawasaki Y., Zhang L., Cheng J.K., Ji R.R. (2008). Cytokine mechanisms of central sensitization: distinct and overlapping role of Interleukin-1 , Interleukin-6, and tumor necrosis factor- in regulating synaptic and neuronal activity in the superficial spinal cord. J Neurosci.

[14] Si H., Yang T., Zeng Y., Zhou Z., Pei F., Lu Y. (2017). Correlations between inflammatory cytokines, muscle damage markers and acute postoperative pain following primary total knee arthroplasty. BMC Muscoskel Disord.

[15] Nandi M., Schreiber K.L., Martel M.O., Cornelius M., Campbell C.M., Haythornthwaite J.A. (2019). Sex differences in negative affect and postoperative pain in patients undergoing total knee arthroplasty. Biol Sex Differ.

[16] Bartley E.J., Fillingim R.B. (2013). Sex differences in pain: a brief review of clinical and experimental findings. Br J Anaesth.

[17] Craft R.M. (2007). Modulation of pain by estrogens. Pain.

[18] Petterson S.C., Raisis L., Bodenstab A., Snyder-Mackler L. (2007). Disease-specific gender differences among total knee arthroplasty candidates. J Bone Joint Surg.

[19] Niki Y., Katsuyama E., Takeda Y., Enomoto H., Toyama Y., Suda Y. (2014). Comparison of postoperative morbidity between simultaneous bilateral and staged bilateral total knee arthroplasties: serological perspective and clinical consequences. J Arthroplasty.

