# Supplementary material for: Postoperative Pain and Opiate Requirement is Increased Following Second-Side Surgery Among Patients Undergoing Staged Total Knee Arthroplasty
Source: Arthroplast Today. 2024 Dec 14;31:101591. doi: 10.1016/j.artd.2024.101591 (PMC11714693; doi:10.1016/j.artd.2024.101591)
Supplement: Conflict of Interest Statement for Emerson [file mmc2.pdf]

CONFLICT OF INTEREST STATEMENT

American Association of Hip and Knee Surgeons

(Adopted from the American Academy of Orthopaedic Surgeons disclosure statement)

The following form **must be filled out completely and submitted by each author (example, 6 authors, 6 forms). All items require a response. If there is no relevant disclosure for a given item, enter "*None*."**

Manuscript Title: **Postoperative Pain and Opiate Requirement is Increased following Second-Side Surgery among Patients undergoing Staged Total Knee Arthroplasty (TKA)**

1. Royalties from a company or supplier (The following conflicts were disclosed)
None

2. Speakers bureau/paid presentations for a company or supplier (The following conflicts were disclosed)
None

3A. Paid employee for a company or supplier (The following conflicts were disclosed)
None

3B. Paid consultant for a company or supplier (The following conflicts were disclosed)
None

3C. Unpaid consultants for a company or supplier (The following conflicts were disclosed)
None

4. Stock or stock options in a company or supplier (The following conflicts were disclosed)
None

5. Research support from a company or supplier as a Principal Investigator (The following conflicts were disclosed)
None

6. Other financial or material support from a company or supplier (The following conflicts were disclosed)
None

7. Royalties, financial or material support from publishers (The following conflicts were disclosed)
None

8. Medical/Orthopaedic publications editorial/governing board (The following conflicts were disclosed)
None

9. Board member/committee appointments for a society (The following conflicts were disclosed)
None

Each author must sign AND print or type his/her name, date and submit a separate form

In addition, one BLINDED Conflict of Interest form (no author names used) should be submitted per manuscript with all author disclosures.

Roger H. Emerson, M.D.	[signature]	8/26/2024
Author Name (Print or Type)	Author Signature	Date
